# Supplementary material for: CBL/CAP Is Essential for Mitochondria Respiration Complex I Assembly and Bioenergetics Efficiency in Muscle Cells
Source: Int J Mol Sci. 2023 Feb 8;24(4):3399. doi: 10.3390/ijms24043399 (PMC9964740; doi:10.3390/ijms24043399)
Supplement: Supplementary file 1 [file ijms-24-03399-s001.zip › ijms-2174442-SI.pdf]

SUPPLEMENTARY MATERIAL

CBL/CAP is essential for mitochondria respiration complex I assembly and bioenergetics efficiency in muscle cells

Cho-Cho Aye, Dean E. Hammond, Sergio Rodriguez-Cuenca, Mary K. Doherty,Phillip D. Whitfield, Marie M. Phelan, Chenjing Yang,Rafael Perez-Perez,Xiaoxin Li, Angels Diaz-Ramos, Gopal Peddinti, Matej Oresic Antonio Vidal-Puig, Antonio Zorzano, Cristina Ugalde and Silvia Mora

Supplementary Figures:

Figure S1

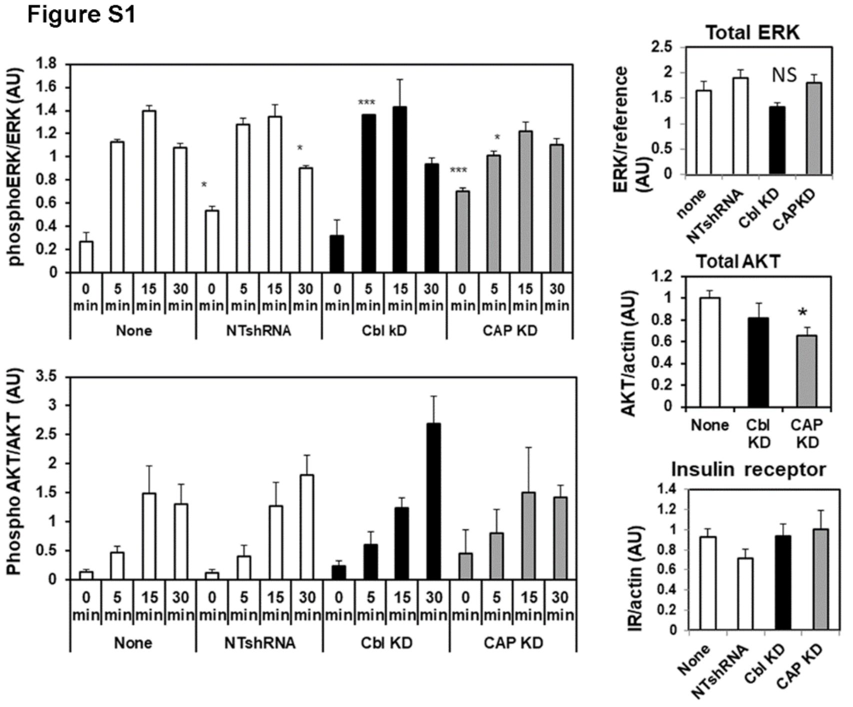

Figure S1. Insulin signaling pathways in control, CBL and CAP depleted cells. Cell lysates were obtained from C2C12 myotubes non-stimulated (0) or treated with 100 nM insulin for 5, 15 or 30 minutes. Equal amount of proteins were immunoblotted with phospho-AKT (S473) or AKT, phospho-ERK, total ERK, insulin receptor and actin. Graphs show mean± SEM of 3 independent experiments quantified and expressed as ratio of PERK/total ERK or pAKT/AKT (arbitrary units). The graphs on the right show the abundance of Insulin receptor, total AKT and total ERK normalized to actin (arbitrary units). Statistical Analysis: one way ANOVA to control non-transfected cells * indicates $p<0.05$, *** $p<0.001$.

Figure S2

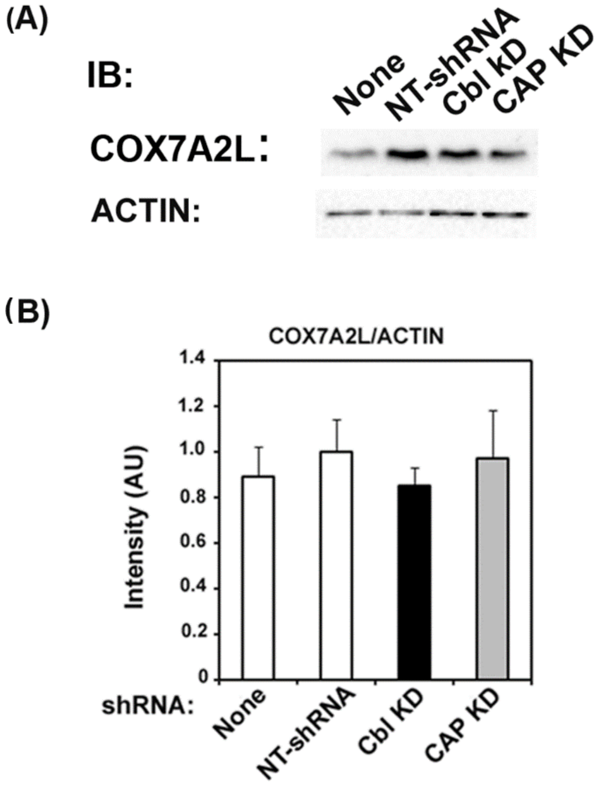

Figure S2. Levels of COX7AL2, in control, CBL and CAP KD cells. Cellular lysates from control untransfected cells (none), cells expressing NTshRNAs, CBL KD or CAP KD cells were immunoblotted with antibodies for COX7AL2 and actin as loading control. (A) shows a representative blot (B) quantification of data from 3 independent experiments, graph show mean± SEM. Graphs show mean±SEM. Statistical Analysis: non significant.

Figure S3

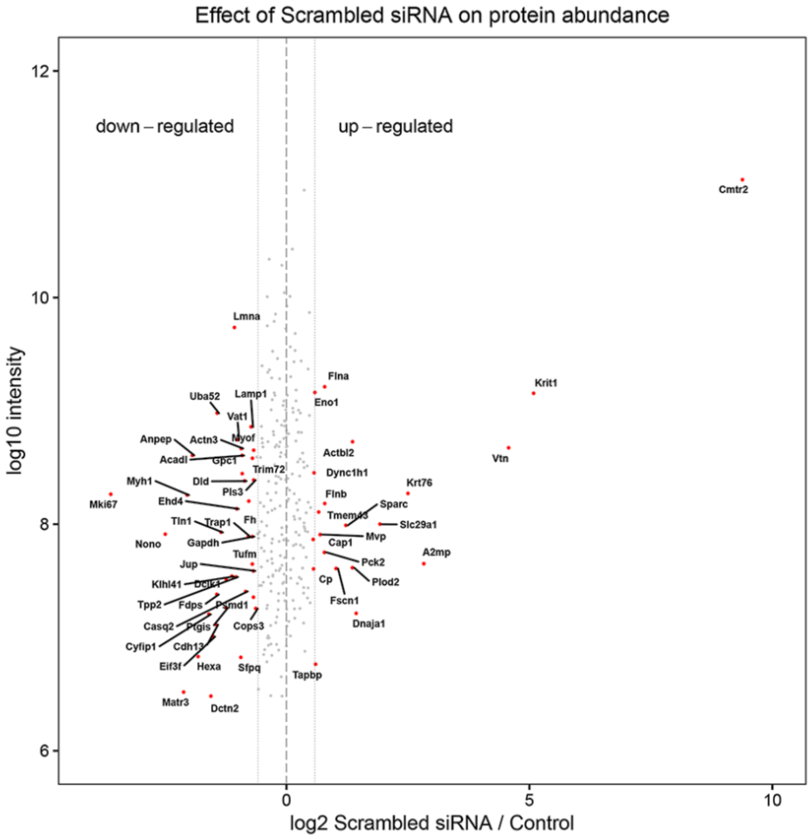

Figure S3. SILAC Proteome comparison of NTshRNA to untransfected control cells. Cells were labelled with heavy stable isotopes of Arg and Lys aminoacids as described in the methods section. Relative abundance of differentially expressed proteins. Those highlighted in red show 1.5-fold or greater differential expression.

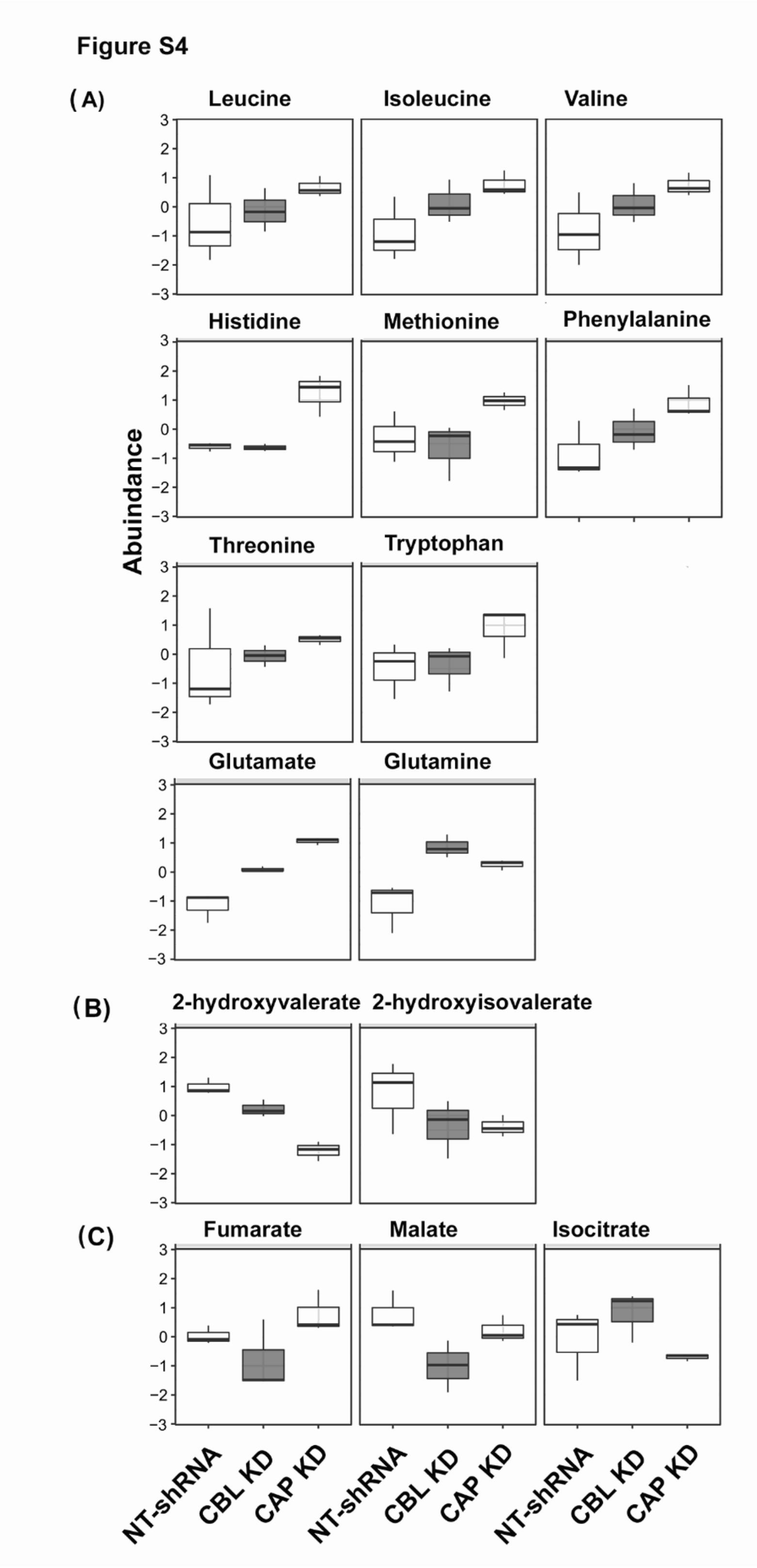

Figure S4. Boxplots of cellular metabolite abundance in control, CBL and CAP knockdown cells. Cellular lysates were analysed by 1H-NMR. Where more than one metabolite spectra were identified a representative of methyl group was selected with a singlet peak and with no/minimal peak overlap. (A) aminoacids (B) Branched amminoacids degradation pathway intermediaries (C) TCA intermediaries.

Figure S5

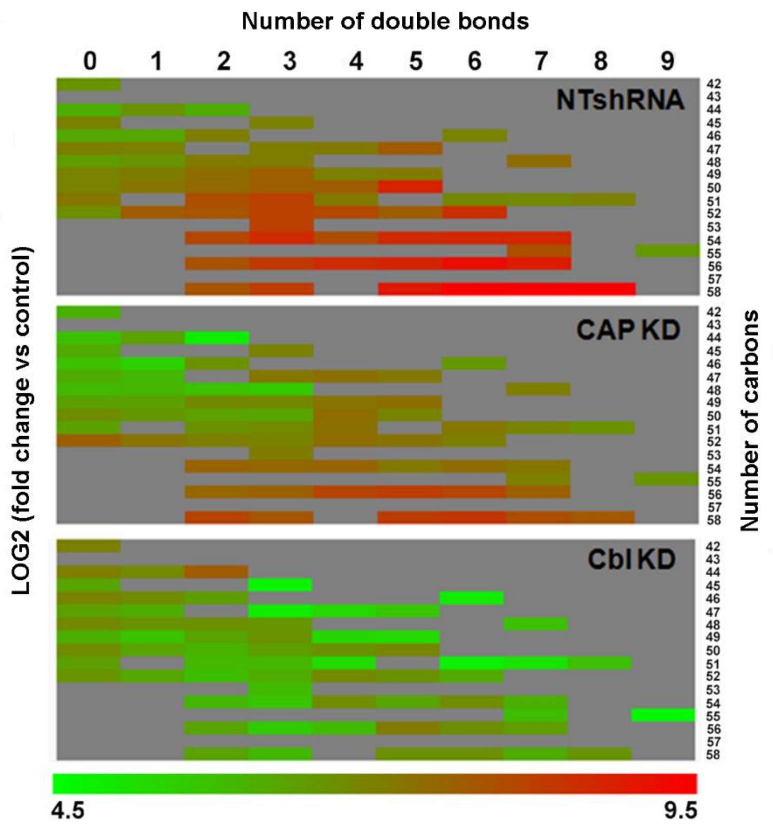

Figure S5. Heatmap of abundance and esterification in triacylglycerols in control, CBL and CAP knockdown cells. Heatmap for TG length and unsaturation index in control, CAP KD and CBL KD, shown as log2 conversions of data relative to control (log2 100 = 6.6). Value > 6.6 and < 6.6 denotes upregulation (red) and downregulation (green), respectively, compared with controls. Pearson correlation for TG length and unsaturation index expressed as fold change relative to control (100%):C length: NTshRNA Pearson: 0.753, p=0.01; CAP KD Pearson 0.800, p=0.01; CBL KD Pearson: -0.139, p>0.05; C unsaturation: NTshRNA Pearson 0.547, p=0,01; CAP KD Pearson 0.454, p=0.01; CBL KD Pearson -0.291, p=0.05.

Supplementary Table S1. Primer sequences used in this study. All primers were obtained from Merck- Sigma- Aldrich.

Name of target	Forward primer: 5’-3’	Reverse primer: 5’-3’
Pgc1a	AACCACACCCACAGGATCAGA	CTCTTCGCTTTATTGCTCCATG
Pgc1b	GGCCTTGTGTCAAGGTGGAT	GGTGCTTATGCAGTTCCGTAC
Nrf1	GAACTGCCAACCACAGTCAC	TTTGTTCCACCTCTCCATCA
Tfam	CCGAAGTGTTTTTCCAGCAT	GGCTGCAATTTTCCTAACCA
Acsl1	GTATGCCACCAGGCCTAAGG	CAGTGGTACCCGCTATTTCCA
18S	TCAAGAACGAAAGTCGCAGG	GGACATCTAAGGGCATCACA
CyclophillinA	GCATACAGGTCCTGGCATCT	TTACAGGACATTGCGAGCAG
Acsl3	TCTTGCAAACAAAGCTGAAGGA	AGGCTTCCCATCAACAGTAATGA
Acsl4	ACCATTGCCATTTTCTGTGAGA	GTGGCATATAAAGTCACAAGTGGAA
Acsl5	TCCTCAAATTTCTGGAGCCTATCT	TGAGCCAAGGGAAGGTAGGAT
Acsl6	CTCCTGAAGCAGTCGGAAGAA	TGCAACCCCCAATCACAGA;
Cpt1	CCAGGCTACAGTGGGACATT	GAACTTGCCCATGTCCTTGT

Supplementary Table S2. **Lipid analysis of cellular lysates by mass spectrometry**. Data are the mean + SDEV of n=3 biological replicates per group. Statistical analysis: multivariate ANOVA with Bonferroni post hoc test: * indicates $p<0.05$ vs Control; ^{T} indicates $p<0.05$ vs NTshRNA (Scramble), Cer: Ceramides; SM: sphingomyelin; Lyso PC: Lysophosphatidylcholines; LysoPE: lysophosphatidylethanolamine; PC: phosphocholine; PE: phosphoethanolamine; TAG: triacylglycerols.

**µmol lipid/µg protein**	**CAP KD**	**Cbl KD**	**No shRNA**	**NTshRNA**
Cer	4.184E-06± 2.24E-07*	3.04E-06± 1.15E-07	3.65E-06± 7.44E-08	5.74E-06± 5.01E-07*
SM	7.216E-06± 4.72E-07	5.44E-06± 1.89E-07	7.74E-06± 6.79E-07	1E-05± 9.44E-07
LysoPC	8.681E-06± 7.97E-07	7.2E-06± 3.7E-07	1.04E-05± 4.66E-07	1.04E-05± 1.15E-06
LysoPCe	3.046E-07± 3.26E-08^{T}	3.08E-07± 8.26E-09	3.85E-07± 2.67E-08	5.74E-07± 1.03E-07
LysoPE	3.568E-07± 3.15E-08^{T}	**2.17E-**07± 1.28E-08*	3.93E-07± 1.82E-08	3.54E-07± 4.2E-08*
PC	4.841E-05± 3.13E-06	**4.37E-**05± 2.62E-06*^{T}	4.93E-05± 1.77E-06	6.17E-05± 5.83E-06
PE	5.643E-05± 6.63E-06	4.13E-05± 4.61E-06	6.57E-05± 7.62E-06	7.68E-05± 6.4E-06
total etherlipids	2.084E-05± 2.8E-06*	1.74E-05± 1.39E-06	2.69E-05± 2.81E-06	2.98E-05± 2.99E-06
TAG	5.724E-06± 2.49E-07^{T}	**4.77E-06±** 3.03E-07^{T}	5.79E-06± 2.95E-07^{T}	9.55E-06± 6.86E-07
(SM+Cer+PC+PE)/TAG	23.91842± 1.238694	23.36812± 3.048135	26.46393± 0.588067	19.27143± 0.950737
unknown	5.154E-05± 1.72E-06	4.34E-05± 4.03E-07	5.54E-05± 1.9E-06	6.46E-05± 6.35E-06
total	0.0002037±	0.000167±	0.000226±	0.00027±

Supplementary Materials and Methods

Lipidome analysis. Cells were grown and differentiated to myotubes, trypsinized and washed twice in PBS followed by centrifugation for 5 min at 1500xg and then snap frozen. Lipid extraction and analysis by mass spectrophotometry is described in detail in the supplemental methods section. Briefly, lipids were extracted with a mixture of chloroform and methanol (2:1, 100 µl), and the samples were centrifuged at 10620xg for 3 min. From the separated lower organic solvent layer, 60 µL was taken into a vial insert and mixed with 10 µL of a standard mixture containing 3 labelled lipid species: L-   -lysophosphatidylcholine palmitoyl-D3 (methyl-D3), 1,2-dipalmitoyl-D6-3-sn-glycerophosphatidylcholine (dimethyl-D6) and tripalmitin-1,1,1-^{13}C3 (concentration level 0.4-0.6 µg). The protein content of the cells was determined by using a Pierce Micro BCA Protein Assay Kit (Thermo Fisher Scientific). Lipidomics were performed on a Waters Q-Tof Premier mass spectrometer combined with an Acquity Ultra Performance LC^{TM} (UPLC). The lipidomics data was processed using MZmine 2 software (*mzmine.sourceforge.net)* including alignment of peaks, peak integration, normalization, and peak identification. Lipids were identified using an internal spectral library. Quantification of lipid subspecies was based on peak heights of internal standards.

Metabolome Analysis. C2C12 cells were grown and differentiated to myotubes. Cells were incubated in DMEM media without serum for 2hrs and then either left untreated or treated for 30 min with 100 nM insulin. Cells were then quickly washed 2x in PBS and snap frozen at -80°C until used. Metabolite extraction, NMR acquisition and spectral processing was carried out as we previously described [52]. Spectra were prepared for statistical analysis by normalizing each spectrum using the Probablistic Quotient Normalisation (PQN) method [53] and spectra bucketed according to spectral features or peaks; all peaks both annotated and unknown were included in the bucket table. Metabolite analysis was carried out in MetaboAnalyst 4.0 (www.metaboanalyst.ca)[54, 55] and R/Bioconductor [56].
